# Incidence and Risk Factors for Tractional Macular Detachment after Anti-Vascular Endothelial Growth Factor Agent Pretreatment before Vitrectomy for Complicated Proliferative Diabetic Retinopathy

**DOI:** 10.3390/jcm8111960

**Published:** 2019-11-13

**Authors:** Andrea Russo, Antonio Longo, Teresio Avitabile, Vincenza Bonfiglio, Matteo Fallico, Francesco Boscia, Claudio Furino, Salvatore Cillino, Mario Toro, Robert Rejdak, Katarzyna Nowomiejska, Michele Reibaldi

**Affiliations:** 1 Department of Ophthalmology, University of Catania, 95100 Catania, Italy; ant-longo@libero.it (A.L.); tavitabi@unict.it (T.A.); enzabonfiglio@gmail.com (V.B.); matteofallico@hotmail.com (M.F.); toro.mario@email.it (M.T.); mreibaldi@libero.it (M.R.); 2 Department of Ophthalmology, University of Sassari, 07100 Sassari, Italy; francesco.boscia@uniss.it; 3 Department of Ophthalmology, University of Bari, 70121 Bari, Italy; claudiofurino@gmail.com; 4 Department of Experimental Biomedicine and Clinical Neuroscience, Ophthalmology Section, University of Palermo, 90100 Palermo, Italy; salvatore.cillino@unipa.it; 5 Department of General Ophthalmology, Medical University of Lublin, 20079 Lublin, Poland; robertrejdak@yahoo.com (R.R.); katarzynanowomiejska@umlub.pl (K.N.)

**Keywords:** anti-VEGF, proliferative diabetic retinopathy, tractional macular detachment, vitrectomy

## Abstract

The study’s purpose was to determine the incidence, risk factors, and outcomes of tractional macular detachment after anti-vascular endothelial growth factor (VEGF) pretreatment before vitrectomy for complicated proliferative diabetic retinopathy. Patients who underwent primary vitrectomy for complicated proliferative diabetic retinopathy, from January 2012 to 31 December 2018, were enrolled. Ophthalmic and pre-operative data were extracted from electronic record systems. All eyes with a valuable Optical Coherence Tomography (OCT)performed within 5 days before injection of anti-VEGF and on the day of vitrectomy were included. Multivariable logistic regression showed that significant risk factors for developing tractional macular detachment included days between anti-VEGF and vitrectomy (OR, 0.71 [95% CI 0.65–0.76]; *p* < 0.001), vitreous hemorrhage (OR, 0.23 [95% CI 0.11–0.49]; *p* < 0.001), and age (OR, 1.05 [95% CI 1.02–1.08]; *p* < 0.001). Decision-tree analysis showed that the stronger predictors of tractional macular detachment were the time between anti-VEGF injection and vitrectomy (*p* < 0.001). Secondary predictors were the presence of vitreous hemorrhage (*p* = 0.012) in eyes that underwent vitrectomy between 6 and 10 days after anti-VEGF injection and younger age (*p* = 0.031) in eyes that underwent vitrectomy 10 days after anti-VEGF injection. Tractional macular detachment occurs in 10% of eyes after anti-VEGF injection, the main risk factors being days between anti-VEGF injection and vitrectomy, vitreous hemorrhage, and age.

## 1. Introduction

Diabetic retinopathy (DR) is the leading cause of visual loss in working-age adults in developed countries [1]. In some studies that reported incidence in Italy, the cumulative incidence of DR ranged from 34% to 59% during a four-year period, depending on the age of patient and severity of disease [2,3]. Proliferative diabetic retinopathy (PDR) is the worst stage of DR, and is characterized by the development of retinal neovascularization, which usually leads to serum leakage, hemorrhage, and fibrovascular proliferation in the vitreous–retinal interface. Fibrosis and neovascularization ultimately lead to vision loss due to tractional retinal detachment (TRD) in untreated eyes [4].

Pars plana vitrectomy is broadly considered the gold standard for management of this condition; however, the occurrence of intra-operative hemorrhage when dissecting the epiretinal neovascular membrane can reduce the outcome of surgery both anatomically and functionally. Reiterated bleeding can also prolong operation time and significantly increase the rate of complications [5,6].

The pathophysiological basis of PDR is angiogenesis [7]. Vascular endothelial growth factor (VEGF) has been confirmed to be a crucial driver of vascular permeability, diabetic macular edema, neovascularization and PDR [8,9,10].

Intra-vitreal administration of anti-vascular endothelial growth factor (anti-VEGF) is variably used as an addition to vitrectomy to improve the outcome of surgery by reducing the incidence of intra-operative and post-operative vitreous hemorrhage and to ease the delamination of fibrovascular membranes [11,12,13].

Intra-vitreal injections of anti-VEGF in adjunct to vitrectomy can therefore determine better visual acuity, fewer retinal breaks, fewer endo-diathermy applications, decrease the duration of surgery, and intra-operative bleeding [13,14,15,16,17].

On the other hand, intra-vitreal anti-VEGF has been proved to be able to increase the severity of fibrosis with progression or development of TRD shortly after injection in patients affected by PDR. Similarly, a fibrotic switch has been shown in diabetic fibrovascular proliferative membranes after bevacizumab [18,19,20].

The purpose of this retrospective study was to evaluate the incidence and risk factors of tractional macular detachment (TMD) following intra-vitreal anti-VEGF pretreatment before vitrectomy for complicated PDR.

## 2. Materials and Methods

In this retrospective multicenter cohort study, we included all consecutive patients who had undergone primary pars plana vitrectomy for TRD, fibrous proliferation, or a combination of these, related to active PDR after anti-VEGF pretreatment, from January 2012 to 31 December 2018, at 4 tertiary vitreoretinal centers in Italy: the Eye Clinic of the University of Catania; the Eye Clinic of the University of Sassari; the Eye Clinic of the University of Bari; and the Eye Clinic of the University of Palermo. Anti-VEGF is indicated for the treatment of patients with diabetic macular edema. The “off-label” status of this medication for PDR, and possible systemic and ocular complications, was discussed in detail, and informed consent was obtained from all patients. The study protocol, approved by the institutional review boards of the coordinating center (University of Catania, Catania, Italy, Ethical code 39/724/2011/pol) and the other participating centers, conformed to the tenets of the Declaration of Helsinki.

Inclusion criteria for the use of intra-vitreal anti-VEGF before vitrectomy for the management of PDR included active proliferative diabetic retinopathy, TRD not involving the macula, fibrous proliferation, or a combination of these with associated reduced visual acuity.

Exclusion criteria consisted of dense vitreous hemorrhage preventing visualization of the posterior pole preoperatively and grading of fibrovascular membranes, eyes that had undergone any previous ocular surgery, except uncomplicated cataract surgery, a history of ocular inflammatory disease, patients with other intraocular diseases that may have affected the vitreoretinal surgery, such as retinal vascular disorders, traumatic retinal detachment, uveitis, and congenital vitreoretinopathies, and patients who had undergone any previous intra-vitreal anti-VEGF injections in the previous 6 months.

All patients were divided into two groups according to the presence or not of TMD on the day of vitrectomy. Patients were considered to have a TMD when preretinal membranes exerted traction on the retina resulting in the presence of sub-macular fluid on Spectral-Domain OCT (SD-OCT). Patients were considered to have fibrovascular proliferation when preretinal membranes exerted traction on the retina with or without intraretinal edema but without subretinal fluid on SD-OCT [21].

Only eyes with a valuable OCT performed within 5 days before injection of anti-VEGF and on the day of vitrectomy were included. The interval between anti-VEGF injection and vitrectomy was also assessed.

Only 1 eye of each subject was permitted into the study. If both eyes of 1 patient qualified for enrollment, the most affected eye was selected for the study [21].

Patient demographics, complete medical and ophthalmic history, the most recent hemoglobin A1c level, years affected by diabetes, ophthalmic data including phackic/pseudophakic condition, and laser photocoagulation partial/panphotocoagulation before anti-VEGF injection were drawn from electronic medical records.

In each center, 2 separate investigators qualified in the methods of chart abstraction were committed to the task of reviewing the charts of patients separately. Definitions for key variables and all data abstraction forms were reviewed. Chart abstractors were blinded regarding the aim of the study.

### Statistical Analysis

The total number of anti-VEGF injections and vitrectomy procedures was identified, and the overall incidence rate of TMD was calculated. Using the chi-square or Fisher exact tests for categorical variables, and Mann–Whitney tests for quantitative variables, potential risk factors for TMD were identified in univariate analyses. Odds ratio was also calculated; for the continuous variables, Exp(B) from binary logistic regression was used. Risk factors that were significant at the *p* = 0.2 level, and the univariate analysis was included in the multivariable logistic regression analysis.

We also investigated the predicting capability of variables using a regression based on conditional inference decision trees. Risk models were developed using a decision-tree induction from class labeled training records: the development of TMD was the dependent variable, and the other attributes were the predictor variables; the individual records are the tuples for which the class label is known, as previously described [22].

All differences were considered to be statistically significant at a 5% probability level, and all reported *p* values are 2-sided. Statistical analysis used IBM SPSS Statistics for Windows (Version 21.0; IBM Corp, Armonk, NY, USA)

## 3. Results

**Figure 1:**
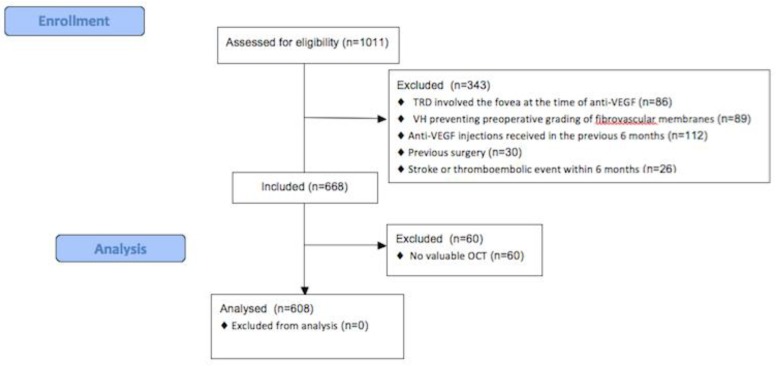
Recruitment flowchart.

The recruitment flowchart is shown in Figure 1.

Overall, charts from 1011 eyes that had anti-VEGF injection before vitrectomy at 4 surgical units between January 2012 and December 2018 were reviewed. 608 eyes were included in the analysis.

The mean ± SD age was 48.7 ± 10.8 years in patients who developed TMD after anti-VEGF injection and 54.8 ± 10.9 years in patients who did not develop TMD after anti-VEGF injection. There were 33/(n = 61) (54.1%) male patients who developed TMD after anti-VEGF injection and 279/(n = 547) (51%) male patients who did not develop TMD after anti-VEGF injection.

### 3.1. TMD Incidence

Overall, the incidence of TMD after anti-VEGF was 10%, 61/608 in the eyes that had anti-VEGF before vitrectomy (chi-square; *p* < 0.001).

### 3.2. Univariate Analysis

Univariate analysis showed that significant variables related with increased risk of TMD included: younger age (48.7 ± 10.8 years in eyes that developed TMD after anti-VEGF, 54.8 ± 10.9 years in in eyes which did not develop TMD; odds ratio [OR], 0.95 [95% CI 0.93–0.97]; *p* < 0.001), higher HbA1c, (7.9 ± 0.7% in eyes that developed TMD, 7.7 ± 0.4% in in eyes that did not develop TMD; OR, 2.12 [95% CI 1.27–3.53]; *p* = 0.004), days between anti-VEGF and vitrectomy (11.8 ± 6.1 days in eyes that developed TMD after anti-VEGF, 5.7 ± 2.7 days in in eyes that did not develop TMD after anti-VEGF; odds ratio [OR], 1.40 [95% CI 1.30–1.52]; *p* < 0.001) (Table 1).

### 3.3. Multivariable Analysis

Significant variables at univariate analysis were taken forward to multivariable analysis. A regression model was constructed for the risk of developing TMD after anti-VEGF injection. Significant risk factors for developing TMD included days between anti-VEGF and vitrectomy (OR, 0.71 [95% CI 0.65–0.76]; *p* < 0.001), vitreous hemorrhage (OR, 0.23 [95% CI 0.11–0.49]; *p* < 0.001), and younger age (OR, 1.05 [95% CI 1.02–1.08]; *p* < 0.001) (Table 2).

### 3.4. Decision-Tree Analysis

**Figure 2:**
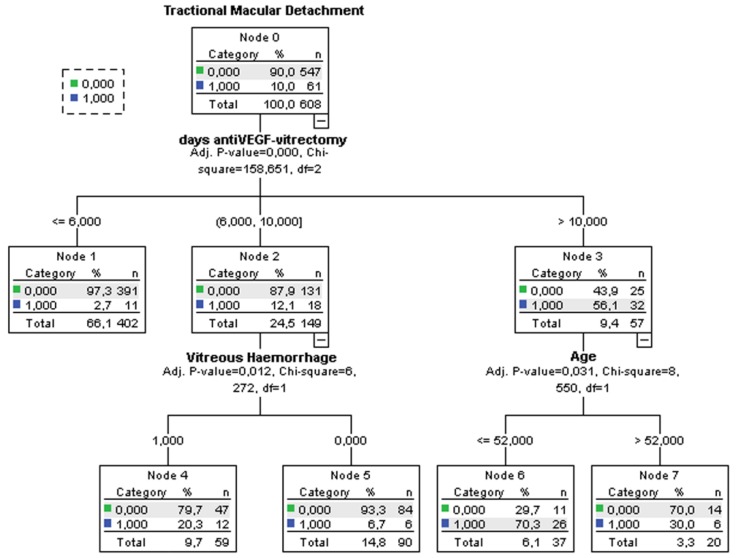
Decision-tree algorithm of the variables associated with tractional macular detachment.

The outcome of the decision-tree analysis for the prediction of TMD is shown in Figure 2.

The most significant prediction factor was the time between anti-VEGF injection and vitrectomy (*p* < 0.001). TMD development is reduced when surgery is performed within 6 days from anti-VEGF injection. Secondary predictors were the presence of vitreous hemorrhage (*p* = 0.012) in eyes that had undergone vitrectomy between 6 and 10 days after anti-VEGF injection and younger age (*p* = 0.031) in eyes that had undergone vitrectomy more than 10 days after anti-VEGF injection.

## 4. Discussion

A challenging condition faced by vitreoretinal surgeons is the surgical management of PDR complications. The most reported post-operative complication after vitrectomy is vitreous hemorrhage, reaching 75% in some studies when pre-operative anti-VEGF was not administered [23,24,25,26].

Neovascularization, secondary to PDR, can effectively be reduced [27,28], and surgical visualization can be improved by pre-operative anti-VEGF, which were shown to reduce intra-operative hemorrhaging and facilitate fibrovascular membrane delamination resulting in a lower incidence of iatrogenic breaks [17,19,20,27,29,30].

However, development or progression of TRD soon after pre-operative anti-VEGF injection may occur in as many as 5.2% of cases [31].

Vitrectomy should therefore be performed after sufficient time has passed to allow regression of neovascularization but before fibrovascular contraction.

Some studies have reported regression of neovascularization during the first week after treatment with intra-vitreal bevacizumab in PDR patients [14,32]. In patients affected by PDR who had anti-VEGF at various intervals before Pars Plana Vitrectomy (PPV), El-Sabagh and associates [30] analyzed different components of proliferative fibrovascular membranes (collagen, CD34, and smooth muscle actin). The vascular component of proliferation was significantly reduced 10 days after intra-vitreal bevacizumab injection, whereas the contractile components were not abundant; therefore, 10 days might be considered the ideal timing for pre-operative intra-vitreal bevacizumab injection. They also showed that pan-endothelial marker CD34, expressed significantly in neovascular vessels, was not significantly reduced until 5 days after intra-vitreal bevacizumab injection, suggesting that pre-operative intra-vitreal bevacizumab administered before 5 days may not provide enough time for neovascular regression.

Van Geest and associates [33] showed that connective tissue growth factor (CTGF) levels were highly correlated with the degree of vitreoretinal fibrosis in PDR. Furthermore, they established that the CTGF/VEGF ratio was the highest predictor of fibrosis in PDR patients and that intra-vitreal anti-VEGF treatment determined augmented fibrosis in PDR patients [33].

Wei and associates [34] showed that the fibrin–fibronectin complex is the key factor that directly promotes the progression of fibrosis, while CTGF increases fibrosis by enhancing the affinity of fibronectin to fibrin in pathologic conditions. They suggested that fibrin–fibronectin complex formation is a molecular mechanism underlying the development of TRD after intra-vitreal anti-VEGF injection. Our results show that the presence of vitreous hemorrhage increases the risk of TRD, therefore it is conceivable that the risk increases with copious vitreous hemorrhage. This might explain why, in our study, the patients diagnosed with dense vitreous hemorrhage were more likely to develop severe TRD after intra-vitreal anti-VEGF injection.

Castillo and associates [21] compared two different pre-operative intra-vitreal bevacizumab treatment intervals in PDR patients undergoing PPV. Pre-operative intra-vitreal bevacizumab performed 5–10 days before PPV produced a significantly better best corrected visual acuity (BCVA) and fewer post-operative complications at 6 months compared to pre-operative intra-vitreal bevacizumab performed 1–3 days before, principally in cases with worse vitreoretinal adhesion and when the indication for surgery was fibrous proliferation, TRD, or a combination of fibrous proliferation, vitreous hemorrhaging, and TRD. Their results suggest that pre-operative intra-vitreal bevacizumab performed 1–3 days before PPV may not allow enough time to effectively regress neovascularization, compared to intra-vitreal bevacizumab performed 5–10 days before PPV [21].

Previous studies reported a pre-operative injection of intra-vitreal bevacizumab from 1 to 33 days before PPV for PDR-related complications [11,14,15,17,19,20,29,30,35,36] and TRD incidence between 5.2% [31] and 9% [37].

The results of our study, performed on over 600 eyes, identified less than 6 days as a safe period to perform PPV following anti-VEGF and an incidence rate of TMD after anti-VEGF of 10%. The possible differences are due to the different inclusion criteria that have been used in various studies. Furthermore, in our study, we evaluated the presence of TMD with OCT examination to have a more sensitive evaluation than just the examination of the fundus.

In our study, multivariate analysis showed that 3 factors had a significant effect on the incidence: days between anti-VEGF and vitrectomy, vitreous hemorrhage, and age. We used a decision-tree analysis to analyze the interactions and priorities among them; this is a modeling procedure that has several advantages, including automatically capturing multilevel interactions among predictors, handling nonlinear relationships, rule generation, and ease of visualization and interpretation [38]. In addition, a decision-tree model can be successfully used in clinical settings since the primary related factors can be identified [39].

The decision-tree model identified the time between anti-VEGF injection and vitrectomy as the main variable associated with TMD.

In eyes where vitrectomy was performed within 6 days of anti-VEGF injection, the risk of developing TMD was extremely low, regardless of other variables. TMD developed in 12% of patients who received vitrectomy between 6 and 10 days after anti-VEGF injection; in this group, the risk significantly increased if there was a vitreous hemorrhage associated with Proliferative Diabetic Retinopathy (PDR). Finally, in the eyes where vitrectomy was performed more than 10 days after anti-VEGF, the risk of developing TMD was very high (over half of the eyes), particularly in patients with a younger age. Therefore, it can be useful to perform surgery within 10 days from anti-VEGF injection or within 6 days in the presence of vitreous hemorrhage.

This study has some limitations. First, some bias in the identification of variables might be introduced by the retrospective nature of the study; however, to minimize bias, a rigorous protocol of data extraction was defined at the study planning stage for all variables. Another limitation of the study is that eyes that had vitreous hemorrhages not allowing the exploration of the fundus were excluded. Furthermore, our results do not necessarily apply to the general population, as they were found in 4 Italian tertiary facilities and we did not evaluate association of the treatment outcome on the type or class of antidiabetics that the patients were taking to keep their diabetes under control.

In conclusion, TMD occurs in 10% of eyes after anti-VEGF injection, the main risk factors being days between anti-VEGF injection and vitrectomy, vitreous hemorrhage, and age.

Further prospective studies are needed to evaluate the efficacy of potential strategies in the prevention of TMD.

## Figures and Tables

**Table 1:** Univariate Analysis of Variables in eyes with and without tractional macular detachment detected at OCT after anti-VEGF intra-vitreal injection.

Variables	Eyes with TMD after anti-VEGF n = 61	Eyes without TMD after anti-VEGF n = 547	*p* Value	OR (95% CI)
Age, mean ± SD (years)	48.7 ± 10.8	54.8 ± 10.9	<0.001 ^a^	0.95 (0.93–0.97)
Males, n (%)	33 (54.1)	279 (51.0)	0.686	1.13 (0.67–1.92)
Diabetes type 1, n (%)	4 (6.6)	26 (4.8)	0.758	0.71 (0.24–2.11)
HbA1c, (%)	7.9 ± 0.7	7.7 ± 0.4	0.004 ^a^	2.12 (1.27–3.53)
Length of diabetes, (years)	16.3 ± 4.9	16.3 ± 3.4	0.969	0.99 (0.94–1.06)
Pseudophakic/aphakic, n (%)	18 (33.8)	194 (35.0)	0.397	0.76 (0.43–1.36)
Vitreous hemorrhage	44 (72.1)	301 (55.0)	0.014 ^a^	2.12 (1.18–3.80)
Localized photocoagulation, n (%)	51 (86.6)	385 (70.4)	0.035 ^a^	2.14 (1.06–4.33)
Extensive photocoagulation, n (%)	10 (16.4)	162 (29.6)	0.035 ^a^	0.47 (0.23–0.94)
Days between anti-VEGF and vitrectomy	11.8 ± 6.1	5.7 ± 2.7	<0.001 ^a^	1.40 (1.30–1.52)

SD = standard deviation; OR = odds ratio; CI = confidence interval; IOL = intraocular lens; ^a^ Statistically significant.

**Table 2:** Independently significant risk factors for developing tractional macular detachment detected at OCT after anti-VEGF intra-vitreal injection in the multivariate logistic regression.

Variables	OR	(95% CI)	*p* Value
Days between anti-VEGF and vitrectomy	0.71	0.65–0.76	<0.001
Vitreous Hemorrhage	0.23	0.11–0.49	<0.001
Age	1.05	1.02–1.08	0.001

OR = odds ratio; CI = confidence interval.
